# PReS-FINAL-2028: ANA positivity and a younger age at onset, but not disease category, predict the risk of uveitis in italian children with juvenile idiopathic arthritis

**DOI:** 10.1186/1546-0096-11-S2-P41

**Published:** 2013-12-05

**Authors:** B Schiappapietra, S Calandra, MC Gallo, R De Marco, V Muratore, S Lanni, A Consolaro, A Martini, A Ravelli

**Affiliations:** 1 Pediatria II, IRCCS G.Gaslini, Genoa, Italy; 2 Ophthalmology, IRCCS G.Gaslini, Genoa, Italy; 3 Pediatria, IRCCS Policlinico San Matteo, Pavia, Italy; 4 University, Genoa, Italy

## Introduction

Juvenile idiopathic arthritis (JIA) is the most common cause of chronic anterior uveitis in childhood, and uveitis is the most frequent extra-articular manifestation seen overall in children with JIA. Although it is well-known that ocular involvement is strongly associated with a constellation of particular clinical features, which include the presence of antinuclear antibodies (ANA), young age at disease onset, female sex, and an asymmetric pattern of arthritis, the relative importance of each risk factor is still a matter of debate.

## Objectives

The aim of the study is to investigate the predictive role of the main risk factors for chronic anterior uveitis in Italian children with JIA.

## Methods

The clinical charts of all consecutive JIA patients with the ILAR categories characterized by a distinctive risk of chronic anterior uveitis (persistent oligoarthritis, extended oligoarthritis, rheumatoid factor-negative polyarthritis, psoriatic arthritis and undifferentiated arthritis) followed by study investigators between 1985 and 2012 were reviewed. Patients with at least two ANA positive determinations (title ≥1:160) where considered ANA-positive. Survival analyses, with the first occurrence of uveitis as the event of interest, were conducted by means of the Kaplan-Meier method. Survival curves were compared by the log-rank test. Factors significantly associated with time to uveitis onset were then tested in a Cox proportional hazards regression model.

## Results

A total of 1,260 JIA patients were included in the study. Four patient were excluded due to occurrence of uveitis before onset of JIA. A total of 278 patients (22.1%) developed uveitis. No association was found between the risk of developing uveitis and patient gender (*p *= 0.70) or ILAR category (*p *= 0.49), whereas the risk of developing uveitis was significantly associated with disease onset before 3.5 years (p < 0.0001) and with positive ANA (p < 0.0001). The predictive role of the positive ANA status and of a younger age at onset was confirmed by Cox proportional hazard regression analysis.

## Conclusion

In a large cohort of JIA patients, we found that the risk of uveitis was associated with a positive ANA status and an age at onset ≤ 3.5 years. No association was found between occurrence of uveitis and ILAR category of JIA.

## Disclosure of interest

None declared.

